# Application of negative pressure sealing drainage technology combined with silver ion sterilization nursing solution in the nursing of necrotizing fasciitis

**DOI:** 10.12669/pjms.38.5.5945

**Published:** 2022

**Authors:** Feng Chen, Changqing Liu

**Affiliations:** 1 Feng ChenDepartment of Anorectal, The Affiliated Nanhua Hospital, Hengyang Medical School, University of South China, Hengyang, Hunan Province 421002, P.R. China; 2 Changqing Liu Department of Operating Room, The Affiliated Nanhua Hospital, Hengyang Medical School, University of South China, Hengyang, Hunan Province 421002, P.R. China

**Keywords:** Necrotizing fasciitis, Silver ion sterilization nursing solution, Negative pressure sealing drainage technology, Wound healing time

## Abstract

**Objectives:**

To study the application effect of negative pressure sealing drainage technology combined with silver ion sterilization nursing solution in the nursing of necrotizing fasciitis.

**Methods:**

Medical records of patients with necrotizing fasciitis, treated in our hospital from June 2019 to June 2021, were selected. Patients were retrospectively assigned into two groups based on the debridement method used: debridement with silver ion sterilization nursing solution Group-I, or debridement with negative pressure sealing drainage technology combined with silver ion sterilization nursing solution. Group-II. Wound healing, dressing change times, healing time, treatment cost and patient satisfaction in both groups were statistically compared.

**Results:**

The wound healing rate of patients in Group-II group was higher than that of Group-I on the 5th, 10th and 15th day after operation (P < 0.05). Dressing change times, healing time and treatment cost of patients in the Group-II were lower than those of Group-I (P < 0.05). Patient satisfaction in the Group-II was 91.4% (54 / 59), which was higher than that of Group-I (75.4% (40 / 53) (P < 0.05).

**Conclusions:**

Negative pressure sealing drainage technology combined with silver ion sterilization nursing solution in the nursing of necrotizing fasciitis is effective. It can promote wound healing, shorten the healing time, reduce the times of wound dressing change and treatment cost. It also improves the overall patient satisfaction, making it an efficient method in clinical application.

## INTRODUCTION

Necrotizing fasciitis is a soft tissue infection characterized by extensive and rapid necrosis of subcutaneous tissue and fascia, often accompanied by systemic toxic shock.^1^,^2^ The disease is a mixed infection of a variety of bacteria, mainly aerobic bacteria such as Streptococcus pyogenes and Staphylococcus aureus.^3^ The disease is characterized by the damage to the subcutaneous tissue and fascia only, and does not involve the muscle tissue of the infected site. The occurrence of necrotizing fasciitis results in flake swelling, pain, purulent blood exudation, tissue necrosis and the formation of ulcer wounds, which may seriously affect patient’s quality of life.^4^,^5^

During the treatment, special measures are required to lower the risk of introducing new infection and to insure proper wound healing. Moreover, long-term dressing change may aggravate patient’s condition as well as increase the burden on patients and the healthcare system.^6^ In the past, silver ion sterilization nursing solution was commonly used in the dressing change process to clean and heal the wound.^7^ Recent studies have indicated that the negative pressure sealing drainage technology, a modern noninvasive adjunctive therapy system, is easy in application and results in efficient drainage. It can accelerate wound healing, has good sealing effect and reduces the risk of infection.^8^

The main objective of this study was to comprehensively evaluate the application effect of the negative pressure sealing drainage technology combined with silver ion sterilization nursing solution in patients with necrotizing fasciitis.

## METHODS

Medical records of patients with necrotizing fasciitis treated in our hospital from June 2019 to June 2021 were selected. The study was reviewed and approved by the hospital ethics committee (No. 2021-KY-135, Date: 2021-09-26). Based on the treatment records, patients (n=112, 54 males, 57 females) were retrospectively divided into two groups. Patients that received debridement treatment with silver ion sterilization nursing solution were assigned into Group-I, and patients that received debridement with negative pressure sealing drainage technology combined with silver ion sterilization nursing solution were included in Group-II.

### Inclusion criteria:

- Diagnosed with necrotizing fasciitis and met the corresponding diagnostic criteria;
- Age ranging from 18 to 65 years;
- Treated with debridement and drainage.

### Exclusion criteria:

- Patients suffering from other inflammatory infectious diseases
- Patients who cannot tolerate the treatment
- Patients with incomplete basic data
- Patients with missing clinical test data
- Patients who voluntarily withdrew from the study

According to the records, all the patients in Group-I received a conventional debridement and dressing change. Briefly, the wound was treated with silver ion sterilization nursing solution. Vaseline oil was selected when filling the wound cavity, and sterile gauze was used for drainage at the wound. Then the wound was covered with sterile gauze and wrapped with medical tape. Dressing change was performed every two to three days according to the exudation of the patient’s wound surface. Patients in Group-II were treated by vacuum sealing drainage combined with debridement treatment with silver ion sterilized care liquid. The method involved cutting the foam dressing according to the wound surface of the patient into a suitable shape and placing it in the wound, while maintaining multiple drainage ports in the dressing of the patient, and then suturing the skin and the edge of the dressing. The drainage tube is then connected to the central negative pressure source and the vacuum high pressure drainage bottle. When the vacuum sealing drainage is used, the rinsing tube is retained, and the silver ion sterilizing nursing solution is injected into the irrigation tube every two to three days. The dressing change times are determined according to the actual wound condition of the patient.^9^

Recorded wound healing rates of the two groups at the 5th, 10th and 15th days after operation were statistically compared. The wound healing rate was calculated as follows: wound healing rate = (initial wound area - unhealed area at the time of measurement) / initial wound area * 100%. Dressing change times, healing time and treatment cost of the two groups were statistically compared. Patient’s satisfaction with the received nursing treatment was analyzed statistically based on the patient’s feedback at discharge. Briefly, patients rated their satisfaction as “very satisfied”, “satisfied” and “dissatisfied”. The total satisfaction = (very satisfied + satisfied) / 100%.^10^

### Statistical Analysis:

SPSS 22.0 was used to process the experimental data. The measurement data were in line with the law of normal distribution and homogeneity of variance and were expressed in (x̄ ±s). T-test was used for the comparison between groups before and after the treatment. The count data were expressed in [n (%). The comparison between groups adopted *χ^2^* test. When p < 0.05, the difference was considered statistically significant.

## RESULTS

Medical records of 112 patients met the inclusion criteria of this retrospective study. Of them, 53 patients in Group-I received debridement with silver ion sterilization nursing solution and 59 patients in Group-II received debridement with negative pressure sealing drainage technology combined with silver ion sterilization nursing solution. There was no significant difference in the basic clinical characteristics between the two groups (P > 0.05). Table-I

**Table I:** basic clinical characteristics of patients with debridement in the two groups (n, χ¯±s).

Group	n	Sex (M/F)	Age (years)	Basic diseases		

				Hypertension	Diabetes	Hyperlipidemia
A	53	30/23	47.28±13.79	19	18	16
B	59	34/25	48.91±13.78	20	19	20
*t/x* ^2^		0.012	0.625		0.176	
*P*		0.913	0.533		0.916	

The wound healing rate of patients in the Group-II on the 5th, 10th and 15th day after operation was higher than that of Group-I (P < 0.05) Table-II while the dressing change times, healing time and treatment cost associated with the Group-II treatment were lower than those of Group-I (P < 0.05) Table-III. The nursing satisfaction of Group-II was 91.4% (54 / 59), which was higher than that of Group-I 75.4% (40 / 53) (P < 0.05). Table-IV

**Table II:** comparison of wound healing rate ( χ¯±s).

Group	n	5d after operation	10d after operation	15d after operation
A	53	22.22±5.33	56.32±4.64	78.34±5.34
B	59	25.66±7.29	60.79±8.01	85.12±8.26
*t*		2.862	3.659	5.207
*P*		0.005	*P*<0.001	*P*<0.001

**Table III:** comparison of dressing change times, healing time and treatment cost ( χ¯±s).

Group	n	Dressing change times (Times)	Healing time (d)	Healing time (d)
A	53	8.15±2.93	20.09±4.88	8065.85±1107.40
B	59	6.31±1.98	17.69±4.32	6227.32±693.91
*t*		3.863	2.759	10.392
*P*		*P*<0.001	0.007	*P*<0.001

**Table IV:** comparison of nursing satisfaction [n(%)].

Group	n	Very satisfied	Satisfied	Dissatisfied	Total satisfaction
A	53	22 (41.5)	18 (33.9)	13 (24.6)	40 (75.4)
B	59	32 (54.2)	22 (37.2)	4 (8.6)	54 (91.4)
*χ^2^*					6.831
*P*					0.009

## DISCUSSION

The results of this study show that negative pressure sealing drainage treatment, combined with silver ion sterilization nursing solution debridement, was associated with faster wound healing, less dressing changes, shorter healing time, lower treatment cost and higher nursing satisfaction than the conventional debridement. Qing Chen et al.^11^ studied the efficacy of debridement combined with negative pressure sealing drainage (VSD) in the treatment of hand high-pressure paint injection injury. In hand high-pressure paint injection injury, debridement combined with VSD technology can prevent wound infection and promote the growth of granulation tissue, which is conducive to wound healing. In addition, the study by Yasheng et al.^12^ showed that negative pressure closed drainage not only had a debridement effect, but also had a good clinical effect in the treatment of chronic osteomyelitis. This is consistent with the results of this study.

Closed continuous negative pressure suction technology is a new type of wound debridement and drainage counting. It can create a wound with sufficient drainage, good blood circulation and good granulation tissue growth, and helps to efficiently control local infection. In this study, patients in the Group-II were treated with negative pressure sealing drainage technology in combination with silver ion sterilization nursing solution. The wound healing rate of patients on the 5th, 10th and 15th day after operation was higher than that of Group-I, indicating that negative pressure sealing drainage technology combined with silver ion sterilization nursing solution can effectively promote wound healing.^13^,^14^ C G Huang et al.^15^ in their study observed clinical effect of the negative pressure sealing drainage (VSD) on 30 patients with alkali burn wounds. The results show that the application of VSD technology in clinical alkali burn wounds can effectively promote the removal of residual alkali, reduce further damage to skin tissue, shorten wound healing time, effectively remove inflammatory mediators and reduce the pain of dressing change. It was also associated with the reduced total cost of treatment and improved patient satisfaction. Application of negative pressure sealing drainage technology can fully drain inflammatory exudates by relying on negative pressure, reduce tissue filling, vascular afterload and inter-tissue pressure, improve capillary circulation and blood flow, and improve the oxygen content of local circulation. In addition, in the process of negative pressure sealing drainage, negative pressure can produce pullout effect, which can stimulate the proliferation of granulation tissue, allowing the wound to heal quicker.^16^,^17^ Our study showed that dressing change times, healing time and treatment cost for patients in the Group-II was significantly lower than in Group-I Additionally, in agreement with previous studies, we showed that negative pressure sealing drainage technology was associated with higher patient satisfaction. It further proves the effectiveness of negative pressure sealing drainage technology in the nursing of necrotizing fasciitis.^18^,^19^

### Limitations of the study:

It includes the retrospective nature of the study and small sample size. Further larger-scale studies with larger cohorts of patients are needed confirm our observations.

## CONCLUSION

Negative pressure sealing drainage technology combined with silver ion sterilization nursing solution is very effective in the nursing of patients with necrotizing fasciitis. It can promote the wound healing, shorten the healing time, reduce the times of wound dressing change and treatment cost, and improve the nursing satisfaction of patients. Hence it a recommended technique for treating necrotizing fasciitis.

### Authors’ contributions:

**FC:** Conceived and designed the study.

**CL:** Collected the data and performed the analysis.

**FC:** Was involved in the writing of the manuscript and is responsible for the integrity of the study.

All authors have read and approved the final manuscript.
